# Transmembrane protein 106A is silenced by promoter region hypermethylation and suppresses gastric cancer growth by inducing apoptosis

**DOI:** 10.1111/jcmm.12352

**Published:** 2014-06-28

**Authors:** Dong Xu, Liujing Qu, Jia Hu, Ge Li, Ping Lv, Dalong Ma, Mingzhou Guo, Yingyu Chen

**Affiliations:** a Key Laboratory of Medical Immunology, Ministry of Health, Peking University Health Science CenterBeijing, China; b Peking University Center for Human Disease Genomics, Peking UniversityBeijing, China; c Department of Gastroenterology & Hepatology, Chinese PLA General HospitalBeijing, China

**Keywords:** transmembrane protein 106A, gastric cancer, apoptosis, epigenetic alteration

## Abstract

Inactivation of tumour suppressor genes by promoter methylation plays an important role in the initiation and progression of gastric cancer (GC). Transmembrane 106A gene (*TMEM106A*) encodes a novel protein of previously unknown function. This study analysed the biological functions, epigenetic changes and the clinical significance of *TMEM106A* in GC. Data from experiments indicate that TMEM106A is a type II membrane protein, which is localized to mitochondria and the plasma membrane. *TMEM106A* was down-regulated or silenced by promoter region hypermethylation in GC cell lines, but expressed in normal gastric tissues. Overexpression of TMEM106A suppressed cell growth and induced apoptosis in GC cell lines, and retarded the growth of xenografts in nude mice. These effects were associated with the activation of caspase-2, caspase-9, and caspase-3, cleavage of BID and inactivation of poly (ADP-ribose) polymerase (PARP). In primary GC samples, loss or reduction of TMEM106A expression was associated with promoter region hypermethylation. *TMEM106A* was methylated in 88.6% (93/105) of primary GC and 18.1% (2/11) in cancer adjacent normal tissue samples. Further analysis suggested that *TMEM106A* methylation in primary GCs was significantly correlated with smoking and tumour metastasis. In conclusion, *TMEM106A* is frequently methylated in human GC. The expression of *TMEM106A* is regulated by promoter hypermethylation. *TMEM106A* is a novel functional tumour suppressor in gastric carcinogenesis.

## Introduction

Gastric cancer (GC) is the fourth most common cancer and the second leading cause of cancer-related death worldwide. Despite considerable advances in surgical techniques, clinical diagnostics and new chemotherapy regimens, the clinical outcome of GC remains unsatisfactory [1], mainly because of a poor understanding of the molecular mechanism of GC development. However, epigenetic inactivation of tumour suppressor genes by promoter methylation is recognized to have a crucial role in gastric carcinogenesis and progression [2–4]. The identification of novel promoter hypermethylated genes may provide insights into the mechanisms of inactivation of tumour suppressive pathways and identify tumour markers for GC.

The *TMEM106A* gene on chromosome 17q21.31 encodes TMEM106A, a member of the TMEM106 family, comprising TMEM106A, TMEM106B and TMEM106C. TMEM106B is a transmembrane protein of unknown function, and is a bona fide risk factor for Frontotemporal lobar degeneration, especially in patients with Progranulin mutations [5–11]. TMEM106B is also associated with cognitive impairment in amyotrophic lateral sclerosis [12], and in the pathological presentation of Alzheimer disease [13]. TMEM106B is a type II integral membrane protein, localized in the late endosome/lysosome compartments and is regulated by lysosomal activities [14,15]. Human TMEM106C is a differentially expressed transcript in ankylosing spondylitis [16], and porcine TMEM106C was a positional and functional candidate for arthrogryposis multiplex congenita [17]. Yu *et al*. identified 54 cellular mRNAs, including *TMEM106A*, which that appear to be Cyclin T1-dependent for their induction in activated CD4^+^ T Jurkat T cells [18]. However, no detailed functional study has been reported.

In our human genomics project, we have cloned hundreds of functionally unknown human open reading frames (ORFs) by searching the human Refseq and expressed sequence tag databases in GenBank. Using a cell-based high-throughput assay, we identified several novel genes associated with cell viability [19], including *TMEM106A*. The present study reports that TMEM106A is localized to mitochondria and the plasma membrane. Loss/reduction of *TMEM106A* expression is associated with promoter region hypermethylation in GC. Restoration of *TMEM106A* expression induced GC cell apoptosis and suppressed GC cell growth, suggesting that TMEM106A is a tumour suppressor in GC.

## Materials and methods

### Cell lines and tissue samples

HeLa, MGC803, BGC823, SGC7901 and MKN45 cell lines were cultured in DMEM (Gibco BRL, Rockville, MD, USA), supplemented with 10% foetal bovine serum (FBS; Biochrom, Cambridge, UK). AGS cells were cultured in Ham's F12 nutrient medium with 10% FBS. N87, PHM82 and NUGC3 cells were cultured in RPMI 1640 medium, supplemented with 10% FBS. HGC-27 cells was maintained in minimum essential medium with 10% FBS.

Fresh tissue samples were obtained from 105 cases of GCs (77 male and 28 female patients) from the Chinese PLA General Hospital in Beijing, China. All samples were surgically resected, snap frozen in liquid nitrogen, and stored at −80°C. Among the 105 GC samples, paraffin blocks were available in 49 cases, with matched adjacent non-tumour tissue. The clinicopathological characteristics of these cases are summarized in Table 2. Informed consent was given by all the patients and controls, and the Clinical Research Ethics Committee of the Chinese PLA General Hospital and Peking University Health science Center approved the study protocol.

### Antibodies and reagents

Polyclonal antibodies against TMEM106A were prepared by immunizing rabbits with chemically synthesized TMEM106A peptides (Fig. S1A, rectangle sequences), purified by peptide affinity chromatography *via* CNBr-activated Sepharose™ 4 Fast Flow (GE Healthcare Bio-Sciences AB, Uppsala, Sweden), according to the manufacturer's instructions. Other antibodies used in this study were: anti-β-actin/ACTB and anti-MYC (Sungene Biotech Tianjin, China), anti-PARP (Cell Signaling Technology, Beverly, MA, USA), anti-Bid (Santa Cruz, CA, USA), anti-tBid (ab10640; Abcam, Cambridge, UK) and DyLight 800/DyLight 680-conjugated secondary antibodies against mouse/rabbit IgG (Rockland, ME, USA). z-VAD-fmk (Promega, Madison, WI, USA), Hoechst 33342 and 5-aza-2′-deoxycytidine (5-Aza) (Sigma-Aldrich, St Louis, MI, USA), Cell Counting Kit-8 (Dojindo Laboratories, Kumamoto, Japan) were also used.

### Construction of TMEM106A plasmid and cell transfection

The full-length TMEM106A cDNA was amplified from a normal human tissues cDNA library (Clontech, Mountain View, CA, USA) by PCR with the forward primer (5′-CGTCTGAGGGAACGCTAAGT-3′) and reverse primer (5′-GAATGAGGAGCAGGGAGAG-3′). The purified PCR product was ligated into the pGEM-T Easy vector (Promega), excised by *Eco*RI digestion and subcloned into the *Eco*RI site of pcDNA.3.1/myc-His (-) B to construct the pcDB-TMEM106A or TMEM106A-MYC (C-terminal MYC tag) plasmids. Based on this plasmid, we also constructed the GFP-TMEM106A plasmid. Specific shRNA mediating *TMEM106A* gene knockdown with the targeting sequence (5′-GGCTGGAAATCAAAGTCCACCATGTGCTT-3′) and non-silencing shRNA vector were constructed by Origene Corporation (Rockville, MD, USA). HeLa cells were transfected by electroporation, as described previously [20], and HGC-27, NUGC3 and BGC823 cell lines were transfected using Lipofectamine 2000 (Invitrogen, Carlsbad, CA, USA), according to the manufacturer's protocol.

### Real-Time quantitative PCR analyses

Real-time PCR was performed with an ABI Prism 7000 Sequence Detection System (Applied Biosystems, Foster City, CA, USA), the human Universal Probe Library (UPL) system (Roche, Mannheim, Germany) and Taqman Gene Expression Master mix (Applied Biosystems). Samples were run in triplicate. All samples were normalized against GAPDH using the comparative CT method (ΔΔCT). The primer sequences and the UPL probes were listed in Table 1.

**Table 1:** Primer sequences used in this study

Primer name	Sequence (5′–3′)
Reverse transcription-PCR	
TMEM106A-F	ATGGGTAAGACGTTTTCCCAG
TMEM106A-R	TCATGGTGGGTGAGGGGT
GAPDH-F	GACCACAGTCCATGCCATCAC
GAPDH-R	TCCACCACCCTGTTGCTGTAG
Real-time PCR	
TMEM106A-F	AGAGGCTGAAGCCCAAGC
TMEM106A-R	GAGGTCACCAGGCAGATGAG
GAPDH-F	TCCACTGGCGTCTTCACC
GAPDH-R	GGCAGAGATGATGACCCTTTT
Bisulphite genomic sequencing (BGS)	
TMEM106A-F	GGTGGGTTTTTAGGGGTTGG
TMEM106A-R	CAACAAAAAATATCTTAACCTCC
Methylation-specific PCR (MSP)	
TMEM106A-M-F	GAGTTAAAGTTTGAGGGGAGTGTTC
TMEM106A-M-R	CGTTGTAGTCGTTCGCGGGTAAGG
TMEM106A-U-F	TGAGTTAAAGTTTGAGGGGAGTGTTT
TMEM106A-U-R	TGTTGTAGTTGTTTGTGGGTAAGGTTTT

### Adenoviral vectors

All of the recombinant adenovirus vectors were based on type 5 (E1/E3 deficient) adenovirus. Ad5-null and Ad5-GFP were purchased from SinoGenoMax (Beijing, China). The complete coding sequence of the TMEM106A was subcloned into the BamHI and EcoRI sites of the pShuttle-CMV vector. The expression cassette of TMEM106A was then transferred into the adenoviral backbone vector pAdxsi, and the recombinant clones were confirmed by DNA sequencing. The recombinant viral vector of TMEM166 (Ad5-TMEM166) was linearized by PacI digestion and packaged into HEK293 cells. Viral particles were purified by caesium chloride density gradient centrifugation and titred by TCID50 method. Ad5-EGFP was used to monitor the efficiency of cell infection and those with higher than 70% infection efficiency were used for further experiments.

### Immunofluorescence and confocal microscopy

HeLa cells transfected with pcDB-TMEM106A plasmids were plated on glass coverslips. Twenty four hours after transfection, the cells were fixed with 4% paraformaldehyde and permeabilized with 0.2% Triton X-100. The cells were then incubated with blocking buffer (3% BSA in PBS) and stained with rabbit anti-TMEM106A antibody overnight at 4°C, followed incubation with FITC-goat anti-rabbit IgG. Nuclei were stained with Hoechst 33342 (H33342). The treated cells were observed and documented with an Olympus FV1000 confocal microscope (Olympus, Tokyo, Japan).

To observe membranes containing TMEM106A, HeLa cells were plated on coverslips the day prior to transfection with the indicated plasmids. Twenty four hours later, cells were incubated with mouse anti-MYC antibody or rabbit anti-TMEM106A antibody overnight at 4°C. Then the cells were stained with FITC-conjugated secondary antibodies for 2 hrs at 4°C, fixed with 4% paraformaldehyde, and observed as above.

### 5-aza-2′-deoxycytidine (5-Aza) treatment

Gastric cancer cell lines were split at low density (30% confluence) 12 hrs before treatment, and then treated with 2 μM of the DNA demethylating agent 5-aza-2′-deoxycytidine (5-Aza) for 96 hrs. The treated cells were harvested for DNA and RNA extraction.

### Methylation-specific PCR (MSP)

Genomic DNA from GC cell lines and tissue specimens were prepared using the proteinase-K method [21], and modified by sodium bisulphite treatment, as described previously [22]. MSP primers for TMEM106A were designed (Table 1) and synthesized according to genomic sequences flanking the presumed transcription start sites. The MSP reaction was performed as previously described [21].

### Bisulphite genomic sequencing (BGS)

Bisulphite-treated DNA was subjected to PCR using primers flanking the targeted MSP regions above. Sequencing primers were listed in Table 1. PCR cycle conditions were as follows: 95°C × 5 min.; 35 cycles (95°C × 30 sec., 55°C × 30 sec., 72°C × 40 sec.); 72°C × 5 min. PCR products were cloned into vector pCR2.1, according to the manufacturer's protocol (Invitrogen). Six to ten colonies were randomly chosen and sequenced.

### Immunohistochemistry

Immunohistochemistry was performed on paraffin sections of GC samples using rabbit anti-TMEM106A antibody. Assigning the percentage of positive tumour cells (0, none; 1, <20% of positive staining cells; 2, 20–50% of positive staining cells; 3, >50% of positive staining cells) scored the extent of TMEM106A staining.

### Cell viability assay

Cells infected with Ad5-null or Ad5-TMEM106A were harvested and plated in 96-well plates at 2000 cells per well and incubated at 37°C. Cell viability was analysed using the Cell Counting Kit-8 [23).

### Colony formation assay

Cells were transfected with pcDB-TMEM106A or empty pcDB using lipofectamine 2000. After 48 hrs of transfection, cells were selected with G418 at 0.3 mg/ml for 2 weeks. G418-resistant colonies with ≥50 cells were counted after crystal violet staining.

### Flow cytometry analysis

To detect the efficiency of adenovirus infection, cells were treated with Ad5-GFP at the indicated multiplicity of infection (MOI) for 24 hrs before harvesting and analysis on a FACSCalibur flow cytometer. An FITC-Annexin V staining Detection kit (Biosea Biotechnology, Beijing, China) was used to analyse cell apoptosis, according to the manufacturer's instructions [23].

### Caspase activity assays

Caspase-2, caspase-3, caspase-9 and caspase-12 activities were measured using the Caspase Fluorometric Assay Kit (BioVision, Milpitas, CA, USA). All procedures were carried out according to the manufacturer's instructions.

### Western blot analysis

Total proteins were extracted and measured by the BCA protein assay reagent (Pierce, Rockford, IL, USA). Equal amounts of protein were separated by 12.5% SDS-PAGE and transferred onto polyvinylidene fluoride membranes. The membranes were blocked and incubated with the indicated antibodies. Blots were visualized using an IRDye 800CW-conjugated secondary antibody, and the fluorescence images were obtained using an Odyssey infrared imaging system (LI-COR Biosciences, Lincoln, NE, USA).

### *In vivo* tumourigenicity

A nude mouse xenograft model was established using 6-week-old female BALB/c nude mice (Experimental Animal Center, Peking University Health Sciences Center, Beijing, China). Mice were housed and maintained in a pathogen-free facility, and the Institutional Authority for Laboratory Animal Care of Peking University approved all experimental procedures and protocols. For *in vivo* treatments, HGC-27 cells infected with Ad5-TMEM106A, Ad5-null or mock were injected subcutaneously into the left axilla of BALB/c nude mice in a total volume of 100 μl (4 × 10^6^ cells), respectively. The tumours were measured using callipers every 2 days for 3 weeks. The tumour volume for each mouse was determined (in cubic millimetres) by measuring in two dimensions and calculated as tumour volume = length × (width)^2^/2.

### Statistical analysis

All analyses were performed with the SAS statistical package. Data were presented as the mean ± SD. Statistical analyses was carried out with the Student's *t*-test, the non-parametric tests or the chi-squared test. A *P* < 0.05 was considered statistically significant.

## Results

### Bioinformatics analysis and expression profile of human TMEM106A

**Fig. 1:**
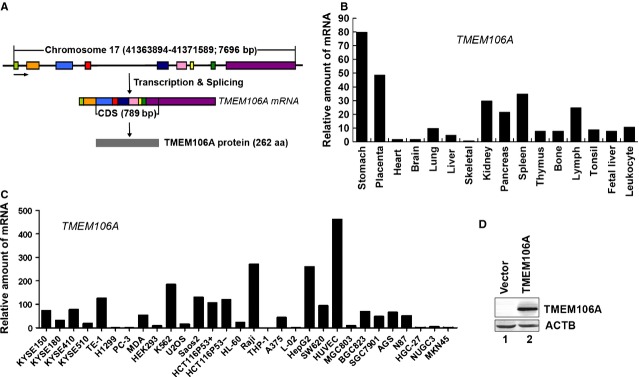
Genetic information and expression profile of TMEM106A. (**A**) Schematic diagram of the gene and mRNA structure of *TMEM106A*. The *TMEM106A* gene is located on chromosome 17, has nine exons, and encodes a protein of 262 amino acid residues. (**B**) *TMEM106A* mRNA expression analysed by quantitative real-time PCR in human normal tissues. (**C**) *TMEM106A* mRNA expression analysed by quantitative real-time PCR in human cell lines. (**D**) Protein expression of TMEM106A in HeLa cells detected by rabbit anti-TMEM106A antibody using Western blotting. ACTB was used as the loading control.

The human *TMEM106A* gene is located on chromosome 17q21.31 and encompasses nine exons and eight introns (Fig. 1A). The full length of human *TMEM106A* cDNA is 2795 bp comprising an ORF encoding a predicted 28.9 kD protein of 262 amino acids (Fig. S1A) with an isoelectric point of 7.04. The alignment of *TMEM106A* sequences from various eukaryotic organisms showed that *TMEM106A* is highly evolutionarily conserved (Fig. S1B and s1C). Transmembrane (TM) analysis (http://www.cbs.dtu.dk/services/TMHMM-2.0/) [24] suggested that TMEM106A is a type II TM protein with a conserved TM domain (94-116 aa) (Fig. S1A, dashed lines). As far as we know, no functional studies have been performed on *TMEM106A*.

The presence of the *TMEM106A* mRNA was confirmed by quantitative real-time PCR and semi-quantitative reverse transcription-PCR in a variety of normal human tissues and cell lines. The *TMEM106A* mRNA is expressed in various tissues and organs (Fig. 1B and Fig. S2A). *TMEM106A* mRNA was detected at a low level or was absent in various cell lines (Fig. 1C and Fig. S2B). The rabbit anti-TMEM106A specific antibody, prepared by using chemically synthesized TMEM106A peptides, was validated by Western blotting (Fig. 1D). From our repeated experiments, we could not detect the expression of endogenous TMEM106A protein.

### TMEM106A is a type II membrane protein and localizes to plasma membrane and mitochondria

**Fig. 2:**
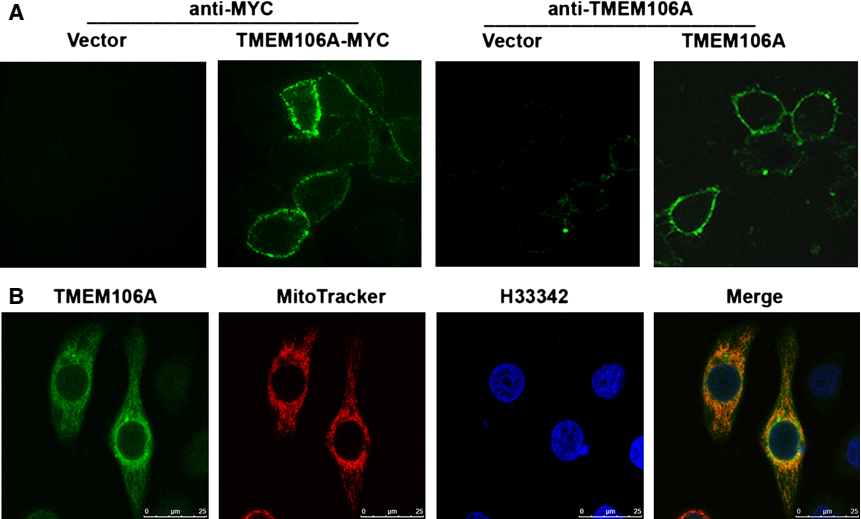
Localization of TMEM106A. (**A**) Immunofluorescence analysis of TMEM106A expression in pcDB-TMEM106A –MYC or pcDB-TMEM106A transfected HeLa cells. Representative images of TMEM106A distribution observed by confocal in live HeLa cells. (**B**) HeLa cells were transiently transfected with pcDB-TMEM106A. Twenty four hours after transfection, cells were fixed, permeabilized and blocked, incubated with rabbit anti-TMEM106A antibody, followed by FITC-goat anti-rabbit IgG and MitoTracker. Nuclei were stained with Hoechst 33342 (Blue) and confocal analysis was performed.

Bioinformatic prediction program suggests TMEM106A is a type II membrane protein that lacks an N-terminal signal peptide and may contain one TM domain. To confirm this prediction, HeLa cells were transfected with empty vector or TMEM106A-MYC (MYC is in the C-terminus) plasmids for 24 hrs, stained with anti-MYC monoclonal antibody, and observed by confocal microscopy. Overexpressed TMEM106A-MYC is localized in the living HeLa cell membrane, with its C-terminus facing extracellular space (Fig. 2A). The membrane-expressed TMEM106A protein was also recognized by the rabbit anti-TMEM106A antibodies (Fig. 2A, right). Consistent with the confocal observation, we also did flow cytometry analysis and found that TMEM106A-MYC appeared in the cell membrane surface in living cells (Fig. S3).

To observe the subcellular localization of TMEM106A, HeLa cells were transfected with pCDB-TMEM106A and marker plasmids (DsRed-ER and DsRed-Golgi) or dye (MitoTracker and LysoTracker) for subcellular compartments stained by immunofluorescence assay, and observed by confocal microscopy. Overexpressed TMEM106A was colocalized predominantly with MitoTracker (Fig. 2B), but not with DsRed-ER, DsRed-Golgi and LysoTracker (data not shown). Taken together, these results indicate that TMEM106A is localized in the plasma membrane and mitochondria.

### *TMEM106A* mRNA is down-regulated or silenced by promoter hypermethylation in GC cell lines

**Fig. 3:**
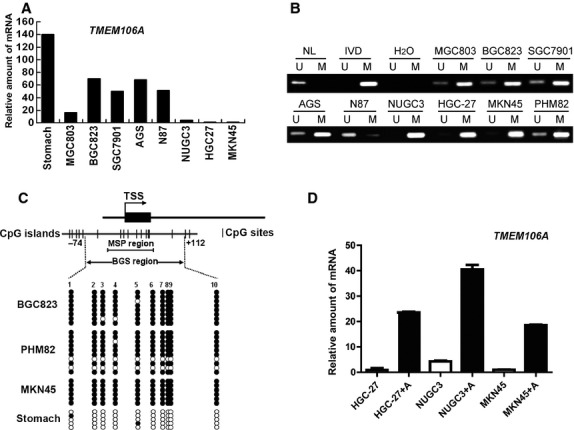
TMEM106A expression was down-regulated or silenced by DNA methylation in GC cell lines. (**A**) *TMEM106A* mRNA expression analysed by quantitative real-time RT-PCR in eight GC cell lines and non-tumour stomach. (**B**) Methylation of TMEM106A determined by MSP. (**C**) A typical CpG island spanning the promoter region of TMEM106A. Each vertical bar represents a single CpG site. A curved arrow indicates the TSS. A region for MSP and BGS is shown. BGS analysis proved the methylation status of TMEM106A in GC cell lines and non-tumour stomach. Filled circles represent methylated CpG sites and open circles denote unmethylated CpG sites. (**D**) *TMEM106A* mRNA expression was restored after treatment with the demethylation reagent 5-Aza (2 μM). Data are presented as mean ± SD. A, 5-Aza (5-aza-2′-deoxycytidine); M, Methylated alleles; U, Unmethylated alleles; NL: Normal blood lymphocyte DNA; TSS: Transcriptional start site; MSP: Methylation-specific PCR; BGS: Bisulphite genomic sequencing.

Our results suggested that *TMEM106A* expression was reduced or absent in most tumour cell lines (Fig. 1C and Fig. S2B). Promoter region methylation is thought to play an important role in gene expression regulation. Bioinformatic analysis indicated that *TMEM106A* contains a typical CpG island in the promoter region. Therefore, we explored the possibility of promoter region methylation and *TMEM106A* mRNA expression. Using real-time quantitative PCR, we detected the levels of *TMEM106A* mRNA in eight GC cell lines and non-tumour stomach tissue. By comparison with non-tumour stomach, the levels of *TMEM106A* mRNA in detected GC cell lines were significantly decreased. Nevertheless, the *TMEM106A* mRNA expressed in these eight cell lines was different. The expression of *TMEM106A* mRNA was almost absent in HGC-27, NUGC3, MKN45 and MGC803 cell lines, while other cell lines such as BGC823, SGC7901, AGS and N87 displayed higher levels of *TMEM106A* mRNA compare to the former (Fig. 3A). Figure 3B was the results of MSP analysis. It was noted that the *TMEM106A* promoter was partially methylated in MGC803, BGC823, SGC7901, AGS, PHM82 and N87 cells, but the methylation level of N87 is the lowest. The almost complete methylation was detected in MKN45, HGC-27 and NUGC3 cells. Comparison of Figure 3A and B, we found that there was a good correlation between expression down-regulation and methylation of *TMEM106A* in these cell lines. That is to say, fully methylated cell lines (MKN45, HGC-27 and NUGC3) almost did not express *TMEM106A* mRNA, whereas partially methylated cell lines (SGC7901, AGS and N87) expressed higher levels of *TMEM106A* mRNA, except for MGC803. To validate the MSP results and further analyse the methylation status of the *TMEM106A* promoter, BGS was performed which confirmed the MSP analysis (Fig. 3C).

We further investigated if *TMEM106A* expression could be rescued by pharmacological demethylation. MKN45, HGC-27 and NUGC3 cell lines were treated with the DNA methyltransferase inhibitor 5-Aza. Results from quantitative PCR assay demonstrated that the re-expression of *TMEM106A* was observed in these cells treated by 5-Aza (Fig. 3D). Taken together, our data suggested that TMEM106A expression is regulated by promoter methylation in GC cells.

### Frequent *TMEM106A* methylation and down-regulation of TMEM106A protein in primary GCs

**Fig. 4:**
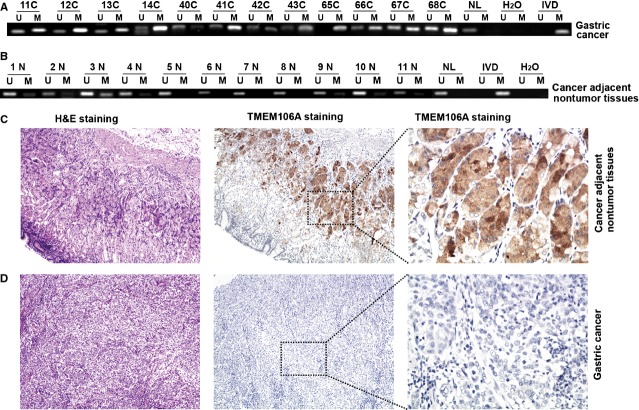
Frequent *TMEM106A* methylation and down-regulation of TMEM106A protein in primary GCs. (**A**) Promoter methylation of *TMEM106A* was analysed by MSP in primary GCs and in cancer adjacent non-tumour tissues (**B**). NL: Normal blood lymphocyte DNA; IVD: *In vitro* methylated DNA; M: Methylated alleles; U: Unmethylated alleles. (**C**) Representative images of TMEM106A protein expression in cancer adjacent non-tumour tissues and their primary GCs (**D**) determined by IHC. Left, HE staining, ×100; Middle and Right, IHC, ×100 and ×400.

To explore the methylation status of *TMEM106A* in primary GCs, 105 cases of GCs were examined by MSP. Frequent methylation of TMEM106A was detected in GCs (93/105, 88.6%; Fig. 4A), while only two cases were methylated among 11 cancer adjacent non-tumour samples (2/11, 18.1%; Fig. 4B). Further analysis suggested that *TMEM106A* methylation in primary GCs was significantly correlated with smoking (*P* = 0.0422), and tumour metastasis (*P* = 0.0462). No association was found with age, gender, alcohol abuse, differentiation and TNM stage (Table 2).

**Table 2:** TMEM106A methylation status and Clinicopathological characteristics in gastric cancer

		*TMEM106A* methylation status		
			
Clinicopathological parameters	*n*	Methylated *n* = 93 (88.6%)	Unmethylated *n* = 12 (11.4%)	*P*-value
Age (years)				
<50	20	17 (85%)	3 (15%)	0.5787
≥50	85	76 (89.4%)	9 (10.6%)	
Gender				
Male	77	69 (89.6%)	8 (10.4%)	0.5808
Female	28	24 (85.7%)	4 (14.3%)	
Alcohol abuse				
Negative	75	68 (90.7%)	7 (9.3%)	0.2883
Positive	30	25 (83.3%)	5 (16.7%)	
Smoking				
Negative	71	66 (93.0%)	5 (7.0%)	0.0422*
Positive	34	27 (79.4%)	7 (20.6%)	
Differentiation				
Poor/Moderate	95	84 (88.4%)	11 (11.67%)	0.5337
Well	3	3 (100.0%)	0 (0.0%)	
T				
T2/T3	7	7 (100.0%)	0 (0.0%)	0.3276
T4	98	86 (87.8%)	12 (12.2%)	
N				
N0	17	13 (76.5%)	4 (23.5%)	0.0882
N1/N2/N3	88	80 (90.9%)	8 (9.1%)	
M				
M0	81	69 (85.2%)	12 (14.8%)	0.0462*
M1	24	24 (100.0%)	0 (0.0%)	
TNM Stage				
II/III	84	72 (85.7%)	12 (14.3%)	0.0670
IV	21	21 (100.0%)	0 (0.0%)	

* *P* < 0.05.

Immunohistochemistry was performed to evaluate TMEM106A protein expression in 49 paired GCs and their cancer adjacent non-tumour tissues. A blinded pathologist scored the expression level of TMEM106A in individual samples. As shown in Figure 4C and D and Table 3, the expression of TMEM106A protein was positive (45/49, 91.8%) in most cancer adjacent non-tumour tissues (Fig. 4C, Table 3), but was near negative (48/49, 98.0%) in primary GCs (Fig. 4D, Table 3).

**Table 3:** Comparison of TMEM106A protein expression in gastric cancer and cancer adjacent tissues

		TMEM106A expression				
			
Groups	*n*	0	1	2	3	*P*-value
Gastric cancer	49	48	1	0	0	<0.0001
Cancer adjacent non-tumour tissues	49	4	11	16	18	

### TMEM106A suppressed GC cell growth

**Fig. 5:**
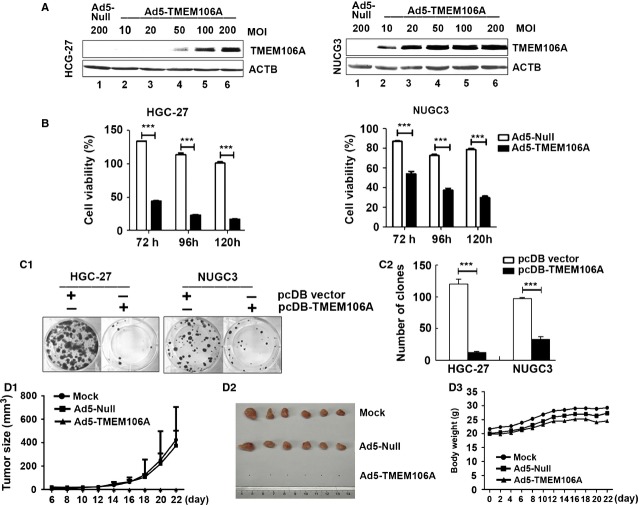
TMEM106A inhibits tumour cell growth. (**A**) HGC-27 and NUGC3 cells were infected with Ad5-TMEM106A at 10, 20, 50, 100, 200 multiplicity of infection (MOI) or Ad5-null at 200 MOI for 24 hrs. The dose-dependent expression of TMEM106A protein was analysed by Western blotting. ACTB was detected as the protein loading control. (**B**) HGC-27 and NUGC3 cells were infected with Ad5-TMEM106A or Ad5-null for indicated time, cell viability was detected by the CCK8 assay. Data are presented as mean ± SD. ***P < 0.001. (**C1**) Representative images of the colony formation in HGC-27 and NUGC3 cells transfected with Ad5-TMEM106A or Ad5-Null were shown. (**C2**) Quantitative analysis of colony numbers is shown for three independent experiments. Data are mean ± SD; ***P < 0.001. (**D**) Ad5-TMEM106A inhibited the growth of tumours *in vivo*. (**D1**) HGC-27 cells infected with Ad5-TMEM106A or Ad5-Null were injected subcutaneously in BALB/c nude mice. Development of tumours (mean volume ± SD) was monitored using callipers every 2 days. (**D2**) Excised xenograft tumours were photographed on day 22. (**D3**) Bodyweight (mean ± SD) was determined over time in different treatment groups.

The frequent silencing of TMEM106A in GC cell lines and primary cancers, but not in normal gastric mucosa, suggested that TMEM106A is a tumour suppressor. Therefore, we examined the effect of ectopic expression of TMEM106A on the growth and viability in HGC-27 and NUGC3 cells with complete methylation and silencing of TMEM106A. Western blotting showed that the TMEM106A protein expression significantly increased in HGC-27 and NUGC3 cells infected with Ad5-TMEM106A (Fig. 5A). We selected MOI 200 for HGC-27 and MOI 50 for NUGC3 for subsequent experiments.

The cell viability of the aforementioned cell lines, infected by Ad5-Null or Ad5-TMEM106A for different times, was assessed using the CCK-8 assay. The growth of HGC-27 and NUGC3 cells infected by Ad5-TMEM106A was inhibited significantly more than with Ad5-Null treatment, and the inhibition was time dependent (Fig. 5B). This effect on GC was further proved by a colony formation assay. TMEM106A re-expression significantly suppressed the colony-forming ability in HGC-27 and NUGC3 cells, compared with the control group (Fig. 5C1 and C2). If *TMEM106A* was silenced in BGC823 cells which were transfected by effective shRNAs against *TMEM106A,* the colony-forming ability was increased compared with shRNA control cells (Fig. S4). Thus, TMEM106A inhibits the growth of GC cells, functioning as a potential tumour suppressor.

On the basis of the above observations, we then tested whether TMEM106A could suppress the growth of GC cells in nude mice *in vivo*. The tumour growth curves of HGC-27 cells infected with mock, Ad5-null or Ad5-TMEM106A alone in nude mice are shown in Figure 5D1. The Ad5-null and mock groups developed grossly visible tumours at the site of injection within 22 days. By comparison, Ad5-TMEM106A displayed smaller tumours that became invisible at the end of the period. At the end of the experiment, xenograft tumours were isolated and photographed (Fig. 5D2). Compared with Ad5-null and mock group, there was no visible tumour in the Ad5-TMEM106A group, indicating TMEM106A inhibits tumourigenicity. In addition, no toxicity was observed in any of the control or treatment groups throughout these studies, as monitored by weight loss (Fig. 5D3).

### TMEM106A induces apoptosis of GC cells *via* the caspase activation

**Fig. 6:**
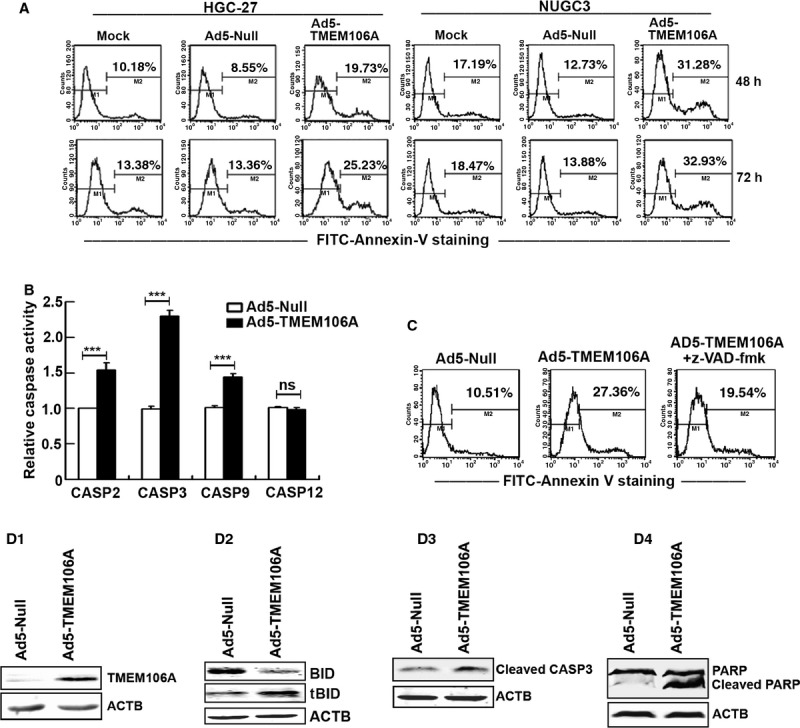
TMEM106A induces apoptosis of GC cells *via* caspase activation. (**A**) HGC-27 and NUGC3 cells were infected with either Ad5-Null or Ad5-TMEM166 for the indicated time. Treated cells were stained with FITC-Annexin V, followed by flow cytometry analysis. (**B**) HGC-27 cells were treated with either Ad5-Null or Ad5-TMEM166 at 200 multiplicity of infection (MOI) for 24 hrs. Caspase-2, Caspase-3, Caspase-9 and Caspase-12 activities were measured using a Caspase Fluorometric Assay Kit and quantified using a POLARSTAR fluorometer. Data are presented as mean ± SD. ***P < 0.001. ns: not significance. (**C**) HGC-27 cells were treated with either Ad5-Null or Ad5-TMEM166 at 200 MOI for 48 hrs or both in the presence or absence of the Z-VAD-fmk (50 μm). Apoptosis was measured by FITC-Annexin V staining, followed by flow cytometry analysis. (**D**) HGC-27 cells were treated with either Ad5-Null or Ad5-TMEM166 at 200 MOI for 48 hrs. Western blotting showing the level of TMEM106A (**D1**), BID and tBID (**D2**), cleavage of caspase-3 (**D3**) and PARP (**D4**). ACTB was detected as the protein loading control.

A key biochemical hallmark of apoptosis is the translocation of phosphatidylserine (PS) from the cytoplasmic surface of the cell membrane to the external cell surface. Exposure of PS on the surface of apoptotic cells can be easily identified by flow cytometry using fluorescence-labelled Annexin V, which specifically binds PS. To characterize TMEM106A-induced growth arrest, we detected PS surface exposure in Ad5-TMEM106A-treated HGC-27 and NUGC3 cells. A representative result of the flow cytometry (Fig. 6A) revealed that HGC-27 and NUGC3 cells treated by Ad5-TMEM106A displayed more apoptotic cells than those cells infected with Ad5-Null.

Caspase-mediated apoptosis is the best-defined cell apoptosis for counteracting tumour growth. To confirm the correlation between the activation of caspase cascades and TMEM106A levels, HGC-27 cells infected with either Ad5-TMEM106A or Ad5-Null were assayed for caspase-2, caspase-3 caspase-9 and caspase-12 activities. The Ad5-TMEM106A-infected cells exhibited elevated caspase-2, caspase-3 and caspase-9 activities compared with Ad5-Null, but there was no obvious difference for caspase-12 (Fig. 6B). To further determine if Ad5-TMEM106A-induced cell apoptosis was caspase dependent, HGC-27 cells were pre-treated with 50 μM z-VAD-fmk for 2 hrs before the addition of Ad5-TMEM106A for another 48 hrs. Annexin V-binding assays showed that pre-treatment with z-VAD-FMK partly decreased cell apoptosis (Fig. 6C), indicating that caspase activation at least partly mediated Ad5-TMEM106A-induced cell apoptosis.

Caspase-2 initiates apoptosis by cleaving the BCL-2 family member BID to form cleaved BID (tBID) [25,26], which results in activation of the mitochondrial pathway and cleavage of caspase-3. Western blotting indicated that compared with the null cells, the overexpression of TMEM106A (Fig. 6D1) in HGC-27 cells markedly decreased the levels of the full-length BID and increased the levels of tBID (Fig. 6D2), following augmented cleaved caspase-3 (Fig. 6D3). Cleaved poly(ADP-ribose) polymerase (PARP), which is associated with caspase-3 activation, was present in Ad5-TMEM106A-infected cells, but rarely detected in control cells (Fig. 6D4). Taken together, these results demonstrated the importance of caspase signalling cascades in Ad5-TMEM106A-mediated cell apoptosis.

## Discussion

We cloned the entire ORF of the human gene transmembrane protein 106A (*TMEM106A*). Sequencing analysis revealed that TMEM106A is conserved in human, chimpanzee, dog, bovine, mouse, rat and rhesus monkey, indicating that it may have important functions in vertebrates. Bioinformatics analysis suggested that TMEM106A is a type II TM protein. TMEM106A is located in the plasma membrane and mitochondria, and is expressed at a low level in various tissues and cell lines. Protein expression of TMEM106A was significantly decreased in primary GC tissues compared with adjacent tissues, suggesting that TMEM106A is important in gastric carcinogenesis.

We showed that the silencing or down-regulation of TMEM106A was closely related with promoter hypermethylation, as demonstrated by methylation-specific PCR, BGS analysis and by the restored expression of TMEM106A in silenced cancer cells obtained using 5-Aza treatment. Thus, promoter hypermethylation of TMEM106A results in transcriptional silencing, which may contribute to the development of GC.

The biological function of TMEM106A in human GC was further investigated *in vitro* and *in vivo*. Ectopic expression of *TMEM106A* in the silenced HGC-27 and NUGC3 cells significantly inhibited cell viability and colony formation ability. In GC cell lines, ectopic expression of TMEM106A induced significant cell apoptosis. We then studied the tumour suppressive effect of TMEM106A against gastric tumour formation *in vivo*: tumour growth was significantly inhibited in nude mice inoculated with HGC-27/Ad5-TMEM106A compared with those inoculated with HGC-27/Ad5-Null. Thus, the *in vitro* and *in vivo* results indicated that TMEM106A functions as a tumour suppressor in gastric carcinogenesis. This anti-tumour effect of TMEM106A in GC suggests that restoration of the function of TMEM106A could halt or reverse GC, thus having a potential therapeutic effect.

The molecular basis of TMEM106A's tumour suppressor property in GC involved activation of the caspase cascade, partly mediated the enhanced apoptosis ability by TMEM106A and subsequent cleavage of substrates. Caspase-2, an initiator caspase, was enhanced by TMEM106A restoration. Caspase-2 is activated by dimerization and then initiates apoptosis *via* the mitochondrial apoptotic pathway [26]. Initiator caspases generally function at the apex of their respective signalling cascades and promote activation of executioner caspases, directly or indirectly. Caspase-2-mediated cleavage of the pro-apoptotic BCL-2 family member BID is generally considered the mode through by which caspase-2 initiates apoptosis [27]. Restoration of TMEM106A activates caspase-2, which activates BID by transforming it into truncated BID (tBID). Once BID is cleaved, the tBID translocates onto mitochondria and promotes mitochondrial membrane permeability and cytochrome c release, thereby triggering the formation of the apoptosome and caspase-9 activation. Activated caspase-9 processes effector caspase-3 to initiate a caspase cascade. Caspase-3 further initiates the proteolytic cleavage of the nuclear enzyme PARP, which causes loss of DNA repair, cellular disassembly and finally, apoptosis. Z-VAD-fmk, a caspase inhibitor, partly rescued cell viability from TMEM106A-induced apoptosis, confirming that activation of the caspase cascade is involved in TMEM106A-induced apoptosis.

To investigate the clinical application of TMEM106A in gastric tumourigenesis *in vivo*, we examined promoter methylation of *TMEM106A* primary GCs and normal controls. TMEM106A gene promoter was methylated in 88.6% of GCs compared with 18.1% of normal controls. If *TMEM106A* is indeed a tumour suppressor, inactivation of the gene by promoter methylation would favour tumour progression and a worse outcome. Therefore, we investigated the clinical significance of *TMEM106A* promoter methylation and its associations with patient clinicopathological factors. *TMEM106A* promoter methylation was significantly associated only with smoking and tumour metastasis. Increasing evidence suggests that promoter methylation could be a predictive biomarker in many human cancers [28–36]. Therefore, *TMEM106A* tumour-specific promoter methylation may become an epigenetic biomarker for GC. Further studies with additional patient cohorts are required to better understand the prognostic significance of TMEM106A in GC.

Gastric cancer is a major health burden worldwide, and conventional treatments for GC (chemotherapy and radiotherapy) have limited effectiveness. Thus, new treatment strategies are needed for GC. Increasingly, therapeutic strategies to inhibit anti-apoptotic signals selectively in tumour cells are being sought that could provide powerful tools to treat GC. Gene therapy is one of the approaches for inducing cancer cell apoptosis, and TMEM106A may show its pro-apoptotic effect in specific and combinatorial treatment approaches in the future.

In conclusion, the novel functional tumour suppressor gene *TMEM106A* is inactivated by promoter methylation in GC, and has important roles in suppressing cell proliferation and inducing apoptosis. TMEM106A induces cancer cell apoptosis through caspase-2/BID activation and the mitochondrial pathway. TMEM106A is a candidate tumour suppressor, and *TMEM106A* methylation may serve as an epigenetic biomarker in GC.

## Supporting information

**Figure S1** TMEM106A is an evolutionarily conserved protein.

**Figure S2** Levels of *TMEM106A* mRNA analysed by reverse transcription-PCR.

**Figure S3** Distribution of TMEM106A.

**Figure S4** Knockdown of TMEM106A increases the colony-forming ability in BGC823 cells.

## References

[1] Moon YW, Jeung HC, Rha SY (2007). Changing patterns of prognosticators during 15-year follow-up of advanced gastric cancer after radical gastrectomy and adjuvant chemotherapy: a 15-year follow-up study at a single Korean institute. Ann Surg Oncol.

[2] Kang GH, Lee HJ, Hwang KS (2003). Aberrant CpG island hypermethylation of chronic gastritis, in relation to aging, gender, intestinal metaplasia, and chronic inflammation. Am J Pathol.

[3] Kang GH, Lee S, Kim JS (2003). Profile of aberrant CpG island methylation along multistep gastric carcinogenesis. Lab Invest.

[4] Lee JH, Park SJ, Abraham SC (2004). Frequent CpG island methylation in precursor lesions and early gastric adenocarcinomas. Oncogene.

[5] Cruchaga C, Graff C, Chiang HH (2011). Association of TMEM106B gene polymorphism with age at onset in granulin mutation carriers and plasma granulin protein levels. Arch Neurol.

[6] Finch N, Carrasquillo M, Baker M (2011). TMEM106B regulates progranulin levels and the penetrance of FTLD in GRN mutation carriers. Neurology.

[7] Van Deerlin VM, Sleiman PMA, Martinez-Lage M (2010). Common variants at 7p21 are associated with frontotemporal lobar degeneration with TDP-43 inclusions. Nat Genet.

[8] Van Der Zee J, Van Langenhove T, Kleinberger G (2011). TMEM106B is associated with frontotemporal lobar degeneration in a clinically diagnosed patient cohort. Brain.

[9] van der Zee J, Van Broeckhoven C (2011). TMEM106B a novel risk factor for frontotemporal lobar degeneration. J Mol Neurosci.

[10] Wood HB (2010). TMEM106B is a susceptibility locus for Ftld. Nat Rev Neurol.

[11] Rollinson S, Mead S, Snowden J (2011). Frontotemporal lobar degeneration genome wide association study replication confirms a risk locus shared with amyotrophic lateral sclerosis. Neurobiol Aging.

[12] Vass R, Ashbridge E, Geser F (2011). Risk genotypes at TMEM106B are associated with cognitive impairment in amyotrophic lateral sclerosis. Acta Neuropathol.

[13] Rutherford NJ, Carrasquillo MM, Li M (2012). TMEM106B risk variant is implicated in the pathologic presentation of Alzheimer disease. Neurology.

[14] Lang CM, Fellerer K, Schwenk BM (2012). Membrane orientation and subcellular localization of transmembrane protein 106B (TMEM106B), a major risk factor for frontotemporal lobar degeneration. J Biol Chemn.

[15] Brady OA, Zheng Y, Murphy K (2013). The frontotemporal lobar degeneration risk factor, TMEM106B, regulates lysosomal morphology and function. Hum Mol Genet.

[16] Assassi S, Reveille JD, Arnett FC (2011). Whole-blood gene expression profiling in ankylosing spondylitis shows upregulation of Toll-like receptor 4 and 5. J Rheumatol.

[17] Genini S, Nguyen T, Malek M (2006). Radiation hybrid mapping of 18 positional and physiological candidate genes for arthrogryposis multiplex congenita on porcine chromosome 5. Anim Gene.

[18] Yu W, Ramakrishnan R, Wang Y (2008). Cyclin T1-dependent genes in activated CD4 + T and macrophage cell lines appear enriched in HIV-1 co-factors. PLoS ONE.

[19] Wang L, Gao X, Gao P (2006). Cell-based screening and validation of human novel genes associated with cell viability. J Biomol Screen.

[20] Chen L, Wang Y, Ma D (2006). Short interfering RNA against the PDCD5 attenuates cell apoptosis and caspase-3 activity induced by Bax overexpression. Apoptosis.

[21] Jia Y, Yang Y, Liu S (2010). SOX17 antagonizes WNT/β-catenin signaling pathway in hepatocellular carcinoma. Epigenetics.

[22] Herman JG, Graff JR, Myöhänen S (1996). Methylation-specific PCR: a novel PCR assay for methylation status of CpG islands. Proc Natl Acad Sci USA.

[23] Chang Y, Li Y, Hu J (2013). Adenovirus vector-mediated expression of TMEM166 inhibits human cancer cell growth by autophagy and apoptosis *in vitro* and *in vivo*. Cancer Lett.

[24] Sonnhammer E, Von Heijne G, Krogh A (1998). A hidden Markov model for predicting transmembrane helices in protein sequences. Proc Int Conf Intell Syst Mol Biol.

[25] Guo Y, Srinivasula SM, Druilhe A (2002). Caspase-2 induces apoptosis by releasing proapoptotic proteins from mitochondria. J Biol Chemn.

[26] Bouchier-Hayes L, Green D (2012). Caspase-2: the orphan caspase. Cell Death Differ.

[27] Boatright KM, Renatus M, Scott FL (2003). A unified model for apical caspase activation. Mol Cell.

[28] Jones PA, Baylin SB (2002). The fundamental role of epigenetic events in cancer. Nat Rev Genet.

[29] House MG, Guo M, Efron DT (2003). Tumor suppressor gene hypermethylation as a predictor of gastric stromal tumor behavior. J Gastrointest Surg.

[30] Issa JPJ (2003). Methylation and prognosis. Clin Cancer Res.

[31] van Rijnsoever M, Elsaleh H, Joseph D (2003). CpG island methylator phenotype is an independent predictor of survival benefit from 5-fluorouracil in stage III colorectal cancer. Clin Cancer Res.

[32] Umetani N, Takeuchi H, Fujimoto A (2004). Epigenetic inactivation of ID4 in colorectal carcinomas correlates with poor differentiation and unfavorable prognosis. Clin Cancer Res.

[33] Guo M, Ren J, House MG (2006). Accumulation of promoter methylation suggests epigenetic progression in squamous cell carcinoma of the esophagus. Clin Cancer Res.

[34] Brock MV, Hooker CM, Ota-Machida E (2008). DNA methylation markers and early recurrence in stage I lung cancer. N Engl J Med.

[35] Park YJ, Claus R, Weichenhan D (2011). Genome-wide epigenetic modifications in cancer. Prog Drug Res.

[36] Blum HE (2011). Gastrointestinal and liver diseases: genetic and epigenetic markers. Gut.

