# Supplementary figures and images for: Transmembrane protein 106A is silenced by promoter region hypermethylation and suppresses gastric cancer growth by inducing apoptosis
Source: J Cell Mol Med. 2014 Jun 28;18(8):1655–66. doi: 10.1111/jcmm.12352 (PMC4190911; doi:10.1111/jcmm.12352)

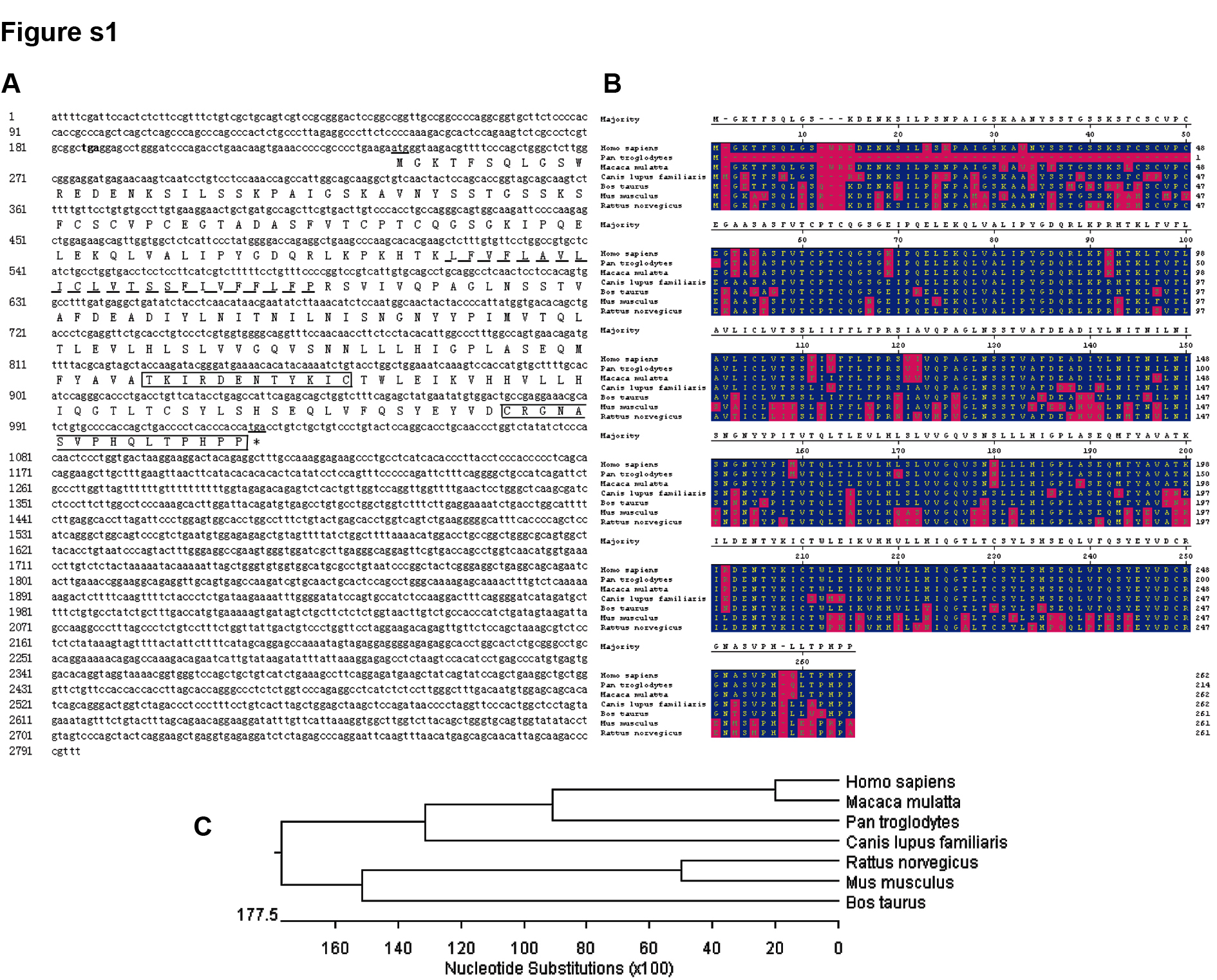

Supplement: Supplementary file 1 — Figure S1 TMEM106A is an evolutionarily conserved protein. [file jcmm0018-1655-SD1.tif]

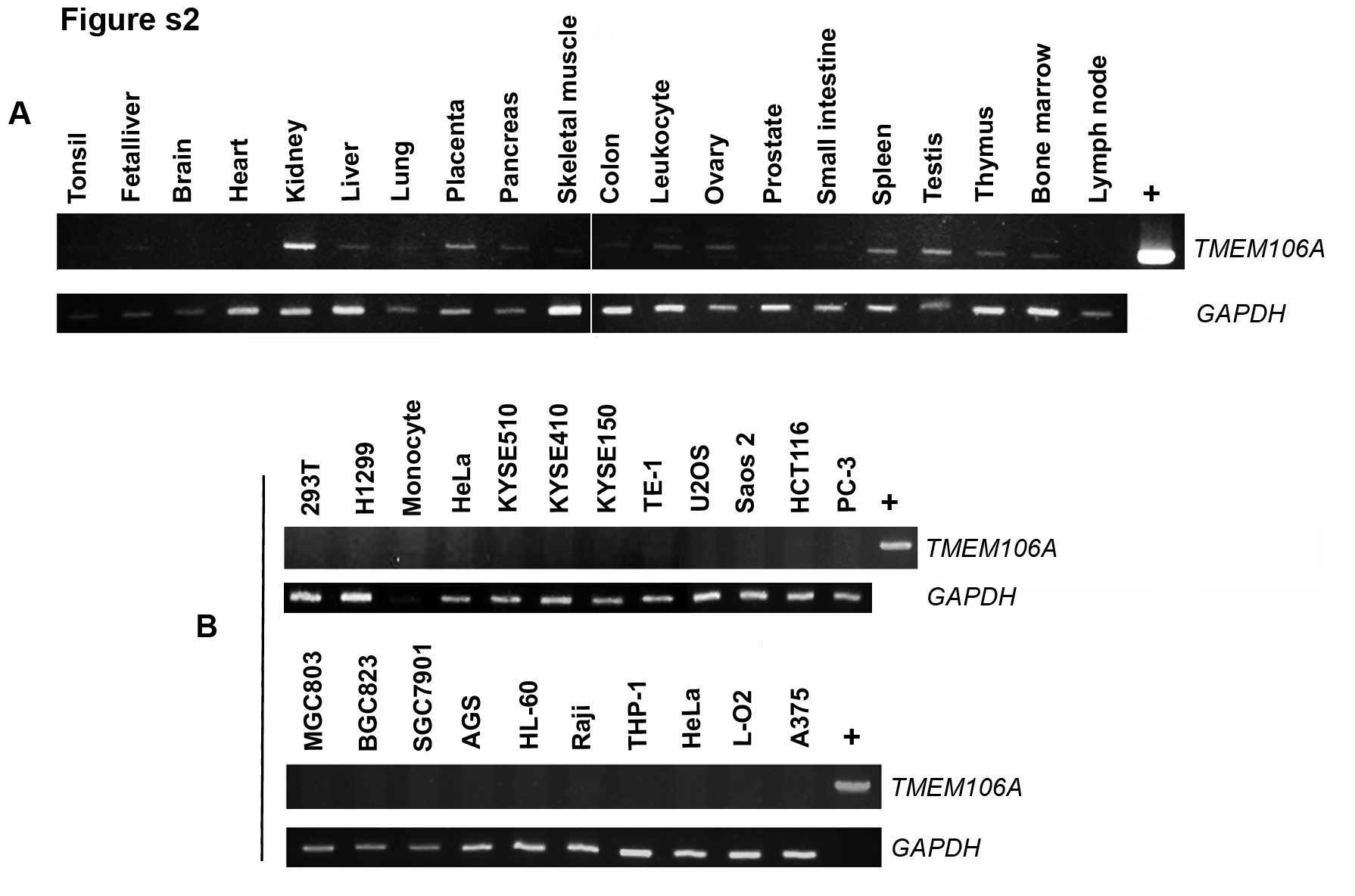

Supplement: Supplementary file 2 — Figure S2 Levels of TMEM106A mRNA analysed by reverse transcription-PCR. [file jcmm0018-1655-SD2.tif]

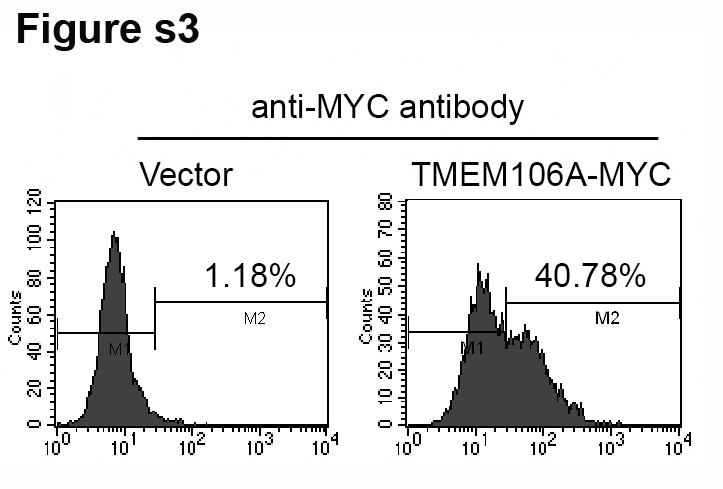

Supplement: Supplementary file 3 — Figure S3 Distribution of TMEM106A. [file jcmm0018-1655-SD3.tif]

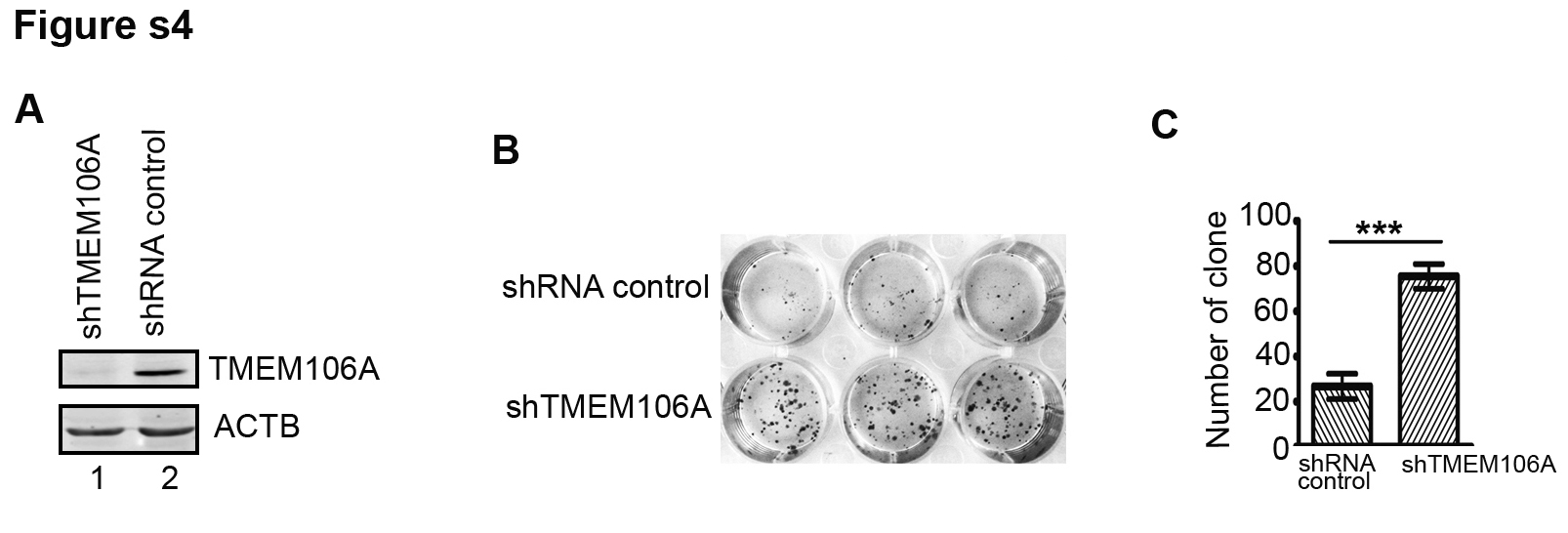

Supplement: Supplementary file 4 — Figure S4 Knockdown of TMEM106A increases the colony-forming ability in BGC823 cells. [file jcmm0018-1655-SD4.tif]
